# Spontaneous regression of metastatic renal cell carcinoma: case report

**DOI:** 10.1186/1752-1947-1-89

**Published:** 2007-09-18

**Authors:** Katerina Lekanidi, Paraskevi A Vlachou, Bruno Morgan, Subramaniam Vasanthan

**Affiliations:** 1 Department of Medicine, Leicester Royal Infirmary, Leicester, LE1 5WW, UK; 2 Department of Radiology, Leicester Royal Infirmary, Leicester, LE1 5WW, UK; 3 Department of Oncology, Leicester Royal Infirmary, Leicester, LE1 5WW, UK

## Abstract

Spontaneous regression of metastatic renal cell carcinoma is rarely observed. A case of suspected spontaneous regression of pulmonary metastases following nephrectomy for histologically proven renal cell carcinoma without systemic treatment is presented along with a brief review of the literature.

## Case presentation

**Figure 1:**
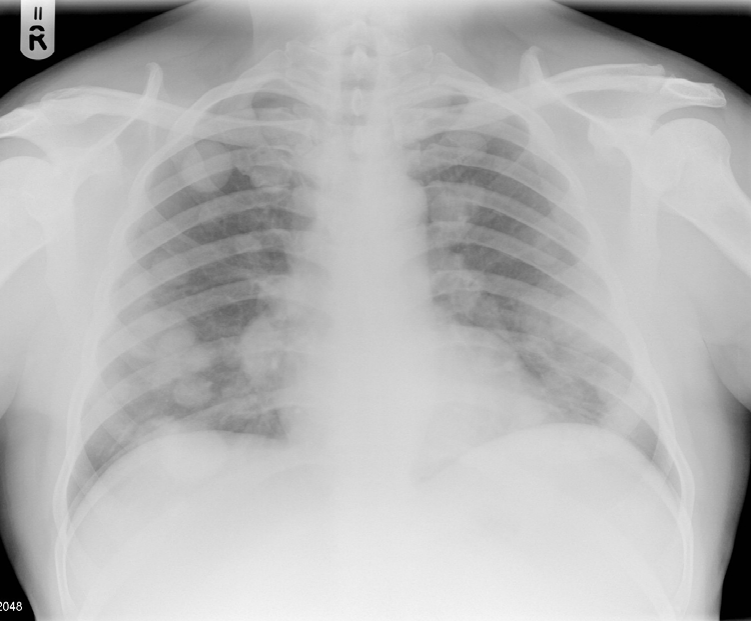
CXR showing multiple bilateral lung metastases at diagnosis.

**Figure 2:**
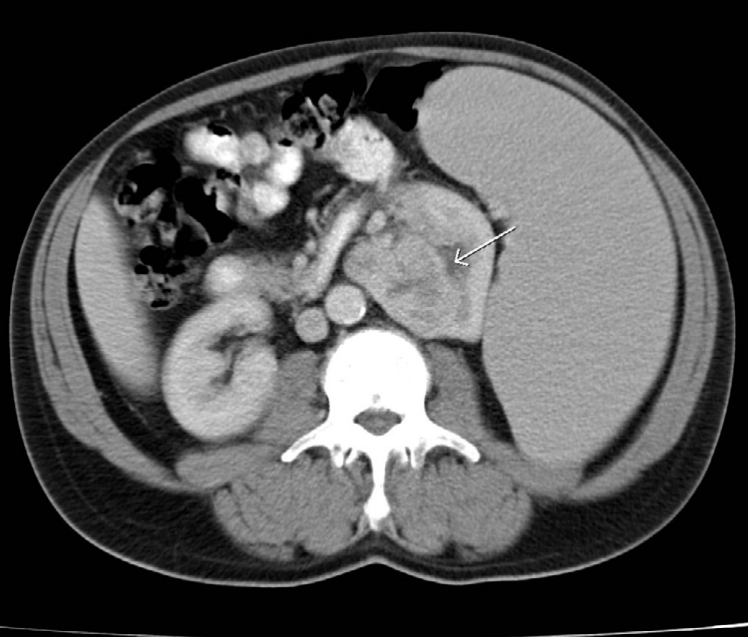
CT showing a left renal carcinoma (white arrow) and splenomegaly.

A 60 year old man, who was under regular haematological follow-up because of myelofibrosis, presented at a routine clinic visit complaining of increasing shortness of breath, weight loss and lethargy. Clinical examination of the chest was normal but a chest x-ray (CXR) showed multiple lung lesions consistent with metastatic deposits (figure 1). A staging computed tomography (CT) scan done shortly afterwards showed marked splenomegaly, causing displacement of the left kidney medially. In the left kidney, there was a 5 cm soft tissue mass arising from the middle of the kidney with characteristics of primary renal cancer (figure 2). The staging CT chest showed multiple pulmonary metastases in both lungs.

**Figure 3:**
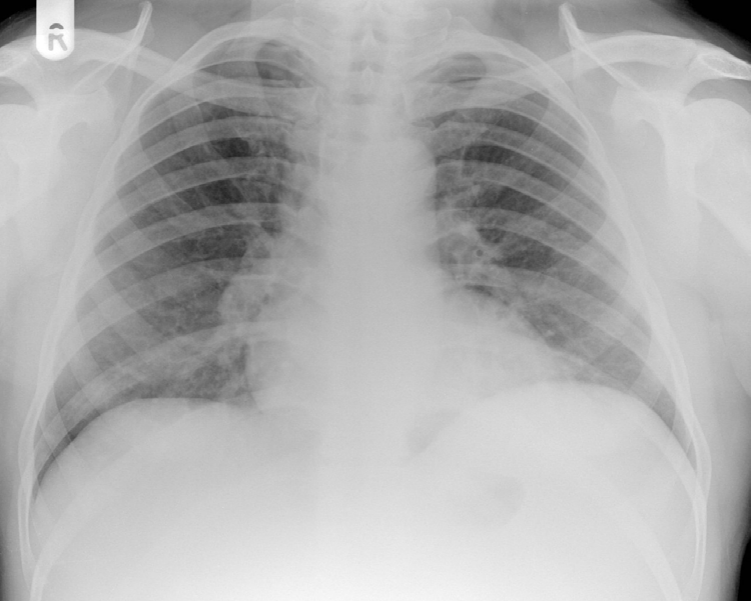
CXR showing no evidence of lung metastases at six weeks after nephrectomy.

The patient underwent laparoscopic cytoreductive nephrectomy without complications and agreed to have immunotherapy with alpha-interferon. Histology revealed clear cell renal cell carcinoma. Six weeks following the operation, just prior to commencing immunotherapy, he attended the haematology clinic as routine follow-up. His initial symptoms had completely resolved and a repeat chest radiograph on that day showed clear lungs with no evidence of metastatic deposits (figure 3). Although no histological confirmation of the metastatic nature of the lung lesions was obtained, it is highly likely that his pulmonary metastases had regressed spontaneously as the patient had not received any immunotherapy in the meantime. The patient remains well five months after the operation.

Renal cell cancer accounts for 2% of all cancers and its incidence is steadily rising. It usually presents in late adult life and is more common in males than females. Although they are associated with Von Hippel-Lindau disease, adult polycystic kidney disease and multicystic nephroma, most renal cell cancers develop spontaneously [1].

The patient may present with urological symptoms such as haematuria or flank pain or with an abdominal mass or alternatively with systemic manifestations, such as anaemia and fever, or symptoms of metastatic disease and other rare phenomena [2].

Approximately 20% to 30% of patients with renal cell carcinoma present with metastatic disease, and 20% to 40% of patients undergoing nephrectomy for clinically localized disease will develop metastasis [3]. If the tumor cannot be completely resected, the course is generally relentlessly progressive, with median survival of 12 to 18 months after metastasis. 85% of relapses occur in the first three years [4].

However, a group of patients with advanced disease have experienced improvements in survival, which is partly related to the introduction of immunotherapeutic approaches and a better understanding of the role and timing of cytoreductive nephrectomy. Although the benefits of immunotherapy have been displayed repeatedly by several studies, controversy has existed as to the need for adjunctive nephrectomy in treating metastatic patients. Removal of the malignant kidney may be of palliative benefit in some settings of metastatic renal cell carcinoma [5]. There have been studies to demonstrate that the benefits of nephrectomy are in addition to and probably greater than the benefit resulting from the interferon-alpha that all patients would receive. Others argue that immunotherapy as a modality has had disappointingly little proven impact on the survival of patients with advanced renal cell carcinoma compared to a variety of other options with less toxicity [6].

There have been case reports in the literature that describe spontaneous regression of metastatic renal cancer [2,7-12].

Bumpus described the first reported case of spontaneous regression of metastatic renal cell carcinoma in 1928 [7]. Metastatic sites include brain, bone, hilar adenopathy and most commonly pulmonary metastases. The clinical pattern of the improvement is not uncommonly the complete disappearance of disease, and often the regression is long-lasting. Many of these cases are associated with surgical removal of the primary tumor, but regression can also occur in association to radiation or embolization of the primary tumor [8].

The rarity of the observation and the heterogeneity of the clinical circumstances in which spontaneous regression of disease occurs do not provide the opportunity for insight into the pathophysiologic mechanism or into the capability for the identification of potential candidates for regression. Although no single mechanism can completely account for this phenomenon, it can be speculated that resection of the primary tumour may result in removal of a prometastatic or growth factor secreted by the tumour and/or promotion of apoptosis might be involved. Immunologic factors almost certainly play a role in some cases of spontaneous tumour regression and perhaps removal of bulk tumour enables or stimulates the immune system to control residual disease. Other theories include hormonal changes, trauma and changes in blood supply (via inhibition of angiogenesis by cytokines) [9].

## Conclusion

It is important to recognize the existence of this clinical entity, which, although rare, might provide another argument in favour of surgical intervention or immunological treatment of metastatic renal cancer. The observation itself should also provide encouragement and drive to pursue immunologic as well as other investigations of the disease.

## Competing interests

The authors declare that they have no competing interests.

## Authors' contributions

SV conceived of the case.

PAV and KL drafted the manuscript.

BM finalized the manuscript.

All authors have read and approved the final manuscript.
